# The Matrons and the College

**Published:** 1917-02-17

**Authors:** 


### THE ROYAL BRITISH COLLEGE OF NURSING.

### THE MATRONS AND THE COLLEGE.

### An Opportunity not to be Missed?

We greatly venture by pointing out to-day, that, the authorities of every hospital, and especially the matrons and lady superintendents of nursing, have a splendid opportunity to help the nurses to help themselves by placing their names upon the Register of the Royal British College of Nursing. It has been stated that some matrons have expressed the feeling that they cannot give any advice or leadership to their own nurses, who must be left to make their own choice and go their own way without interference from the matron or the head of their training-school and teachers, no matter how much the nurses may desire their counsel and guidance. Speaking quite frankly—and we are glad to know this opinion is shared by a large majority of the most experienced and deservedly trusted matrons in this country and in the United States of America—the refusal by a matron or lady superintendent, when consulted, to guide, help, and act as the life-long friend of every nurse, whom it has been her privilege to have under her from the commencement of her nursing career, and whom she has trained and uplifted to the highest standard as a trained nurse, would, in fact, prove destructive to such a matron's reputation and usefulness in the higher positions of the nursing profession. In times like the present, in which the work of trained nurses has been found to be of such inestimable value to the nation and Empire, it is but natural that, every capable and competent nurse should be aroused and filled with the desire to go forward. But her initial step is often difficult from a feeling of diffidence on the part of the nurse, who wishes to turn for counsel to the head of her training-school or to her present matron, whom she regards as a centre of wisdom and judgment, upon whom she justly feels she may rely to help her on her way. How much more has a nurse the right to look to her chief for sympathy and co-operation when the nurses realise they can make their profession a well-organised body, able to act in its own best interests, and also in the interests of all who need their help in the day of sickness, or who may be committed to their care. We have some confidence that there are few, if any, matrons, who are familiar with the great progress made by the Royal British College of Nursing, who will hesitate to give counsel and encouragement to their nurses to join the College Register. Who can doubt that such a step is one best calculated to hasten the hour, when the British Nursing Profession will obtain Statutory Recognition, the Educational Advantages, the Protection and the Status which are so ardently desired by all, who have its best interests at heart? Will the matrons kindly act promptly?

In such circumstances we have thought it well to take the occasion, when through the Manchester meeting, the hollowness and impossibility of the policy of disunion have been tellingly exhibited, through the confession of a member of the Central Committee when face to face with a great meeting of working nurses, to emphasise the fact that, in reality, practically the whole body of British nurses, after full consideration, are ranging themselves as its supporters behind the College. Every matron has a high privilege and honourable responsibilities. She can, if she will, materially aid the completion of the College Register, as a large and increasing number of matrons are doing at the present time, by obtaining application forms for Registration, either from the Editor or direct from the College, and devoting some time and trouble, however much of both it may cost her, to help every nurse under her influence to understand the questions at issue, and to have an opportunity to place her name on the College Register, without avoidable delay. By doing this not only will help be given to individual nurses, but the greatest possible service will be rendered to the profession of nursing in these islands. A general acceptance of this invitation by the matrons of the United Kingdom, whether they be the head of a great or small hospital, a military hospital, a private nursing staff, or an institution for the supply of fully trained nurses, must mean that State Registration, Statutory Recognition, and all the other advantages which can add to the usefulness, position, and authority of the Nursing Profession and its members, will be secured without doubt or avoidable delay. We invite correspondence from our readers, will welcome questions upon any points of difficulty or doubt which may arise, and gladly supply any number of application forms for Registration, to any matron or sister who will send a postcard or a letter to the Editor (28 and 29 Southampton Street, Strand, W.C.), stating exactly the number desired.

